# Supplementary material for: Reconciling Mining with the Conservation of Cave Biodiversity: A Quantitative Baseline to Help Establish Conservation Priorities
Source: PLoS One. 2016 Dec 20;11(12):e0168348. doi: 10.1371/journal.pone.0168348 (PMC5173368; doi:10.1371/journal.pone.0168348)
Supplement: S1 Dataset — (ZIP) [file pone.0168348.s002.zip › Taxa/Serra Norte/SN_2013/N4WS/Lista N4WS-45.pdf]

CAVIDADE N4WS-0045

Classe	Ordem	Fam/Outros	Gên/Outros	Espécie	S-U	U-S	Zon.
Arachnida	Acari	Acaridae		jovem		X	E/P
Arachnida	Araneae	Pisauridae	*Architis*	sp.1	1		P
Arachnida	Araneae	Salticidae		sp.3	X		E
Arachnida	Araneae	Trechaleidae		jovem		1	E
Arachnida	Opiliones	Escadabiidae		jovem	X		P
Arachnida	Opiliones	Sclerosomatidae	*Prionostema*	sp.1	X	X	E
Arachnida	Palpigradi	Eukoeneniidae	*Allokoenenia*	sp.1	X		P
Entognatha	Collembola	Symphypleona		sp.1		X	P
Insecta	Blattodea	Blattellidae		sp.4	1		E
Insecta	Coleoptera	Carabidae		jovem		X	E
Insecta	Coleoptera	Hydrophilidae	Sphaeridiinae	sp.12	X		P
Insecta	Diptera	Fanniidae		sp.		X	P
Insecta	Diptera	Tipulidae	Tipulinae	sp.	X		E
Insecta	Heteroptera	Veliidae	*Paravelia*	sp.1	X		E/P
Insecta	Orthoptera	Phalangopsidae	*Phalangopsis*	sp.	3	2	P
Mammalia	Chiroptera			sp.		3	P
